# Predictability of Pediatric Sepsis Outcome Using SEPSIS-3 Definition in a Single Tertiary Pediatric Institution

**Published:** 2023-06-07

**Authors:** Andrew Rosenzweig, Koichi Yuki

**Affiliations:** 1 Department of Anesthesiology, Critical Care and Pain Medicine, Boston Children’s Hospital, USA; 2 Boston University, USA; 3 Department of Anaesthesia, Harvard Medical School, USA; 4 Department of Immunology, Harvard Medical School, USA; 5 Broad Institute of Harvard and MIT, USA

**Keywords:** Sepsis-3, Pediatric, SOFA

## Abstract

Sepsis is a syndrome of dysregulated response to infection and is associated with high morbidity and mortality. Sepsis was initially defined as a host’s systemic inflammatory response syndrome (SIRS) to infection. In 2016, the importance of dysregulated response was incorporated into the definition of sepsis; adult sepsis was redefined as a life-threatening organ dysfunction caused by a dysregulated host response to infection, with organ function being evaluated by the Sequential Organ Failure Assessment (SOFA) score (Sepsis-3 definition). However, the definition of pediatric sepsis remains the same, based on the original, SIRS-based criteria. In this study, we examined the relationship between mortality and sepsis in pediatric patients in our institution using the Sepsis-3 definition by incorporating the pediatric SOFA (pSOFA) score system, which was reported in 2017. We found that sepsis mortality was better correlated with the pSOFA score in our pediatric cohort. We also found that patients who did not have identified microbes were associated with better survival. In the future, we need to determine the relationship between mortality and Sepsis-3 definition-based pediatric sepsis worldwide to further define the utility of this new definition.

## Introduction

Sepsis is a life-threatening, infection-driven syndrome. Robert C. Boone proposed sepsis as “an invasion of microorganisms and/or their toxins into the bloodstream, along with the organism’s reaction against this invasion” in 1989. Subsequently, sepsis was formally defined as a host’s systemic inflammatory response syndrome (SIRS) to infection in a 1991 consensus conference (Sepsis-1 conference) [1]. In a conference in 2001 (Sepsis-2 conference), the sepsis definition remained the same, and sepsis complicated by organ dysfunction was additionally defined as severe sepsis [2]. However, SIRS can be an appropriate response to infection or any other stimulus that activates the inflammatory process rather than a dysregulation of the host response. The Task Force realized inadequate specificity and sensitivity of the SIRS criteria for observed “sepsis” mortality and therefore the necessity to differentiate sepsis from uncomplicated infection. In 2016, sepsis was defined as a life-threatening organ dysfunction caused by a dysregulated host response to infection (Sepsis-3 conference) [3]. Organ dysfunction was determined by an acute change in total Sequential Organ Failure Assessment (SOFA) score of 2 points or greater caused by an infection [3].

SOFA score system was originally reported in 1996 as a Sepsis-related Organ Failure Assessment score to describe organ dysfunction/failure with the recognition that multiple organ injury is a major cause of morbidity and mortality in critically ill patients [4], but it had not been formally incorporated into the definition of sepsis. This scoring system was later named as Sequential Organ Failure Assessment score to determine the degree of organ injury in general. The score consists of six domains (cardiovascular, respiratory, neurological, coagulation, hepatic, renal). Each domain is given a score ranging from 0 to 4 with the highest total score of 24. However, the original SOFA score system was primarily designed for adult patients because age-dependent organ development was not taken into consideration for the scoring. As a result, in the Sepsis-3 conference, this new sepsis definition was applied to adult patients alone. In adult sepsis, the SOFA score of 2 points or more was associated with an in-hospital mortality greater than 10% [3]. In contrast, pediatric sepsis continues to be defined based on the criteria proposed by Sepsis-1 & Sepsis-2 conferences.

Matics, et al. reported a pediatric SOFA score system (pSOFA) and applied it to patients who were cared for in their institution from 2009 to 2016 [5]. They showed that the maximum pSOFA score correlated well with in-hospital mortality. However, this pSOFA score system has not been applied to the data at other institutions. Our hospital is one of the largest tertiary pediatric institutes in the U.S. In this report, we attempted to apply the Sepsis-3 definition to our pediatric cohort for validation using the pSOFA score system.

## Methods

### Data collection

This retrospective cohort study was approved by the Institutional Review Board (IRB no. IRB-P00041981). Informed consent was waived. We enrolled 806 patients who were admitted to the intensive care unit (ICU) with a diagnosis of sepsis based on the International Classification of Diseases (ICD)-9 and ICD-10 code from January 2014 to December 2019 at Boston Children’s Hospital. Among them, we found 665 patients who were younger than 18-years-old. For those 665 patients, we collected their demographic information and vital signs (oxygen saturation, mean arterial blood pressure) at the ICU, determined the presence or absence of respiratory support, length of stay in the ICU, types of microbes detected if any, laboratory data (complete blood count, chemistry, liver function test, kidney function test, arterial blood gas), and drugs used from the electronic medical record. To assess the organ function, we used the pediatric SOFA system reported by Matics, et al. [5]. The pSOFA system is summarized in Table 1. In our institution, the Glasgow coma scale is not routinely reported unless a patient experiences any neurological issue. Thus, patients who did not have comments on neurological status were considered neurologically intact.

### Statistical analysis

We presented the number and percentage for categorical variables, mean and standard deviation (SD) for continuous variables with normal distribution, or median and interquartile range (IQR) for variables with skewed distribution. The Shapiro-Wilk test was used to assess normality. Potential risk factors for Sepsis-3 vs. non-Sepsis-3 (or mortality) were assessed with univariable logistic regression analysis. The results were shown as odds ratio (OR) and 95% confidence interval (CI). P < 0.05 was considered statistically significant. To determine the cutoff value to predict mortality, Youden-J statistics were used. The statistical analysis was performed using STAT13 (College Station, TX, USA).

## Results

### Sepsis-3 group was associated with higher mortality

Table 2 described the relationship between pediatric patients who met the criteria of Sepsis-3 and who did not. Out of 665 patients we examined, 69 patients did not meet the criteria of Sepsis-3 (non-Sepsis-3 group). This cohort did not have any in-hospital mortality. 572 patients met the criteria of Sepsis-3 (Sepsis-3 group). The mortality was 8.0% in this group. There was no statistically significant difference in age and gender between the two groups. As expected, the maximum subscore for each organ domain was significantly higher in the Sepsis-3 group compared to the non-Sepsis-3 group. This was true for the average subscore. Maximum pSOFA and average pSOFA scores were also higher in the Sepsis-3 group.

### Non-survivors were associated with significantly higher maximum and average pSOFA scores

Table 3 described the relationship between patients who passed away during the stay and who survived. There was no difference in age and gender between the groups. However, the non-survivor group was associated with significantly higher maximum (and average) subscores as well as maximum (and average) pSOFA scores compared to the survivor group.

**Figure 1:**
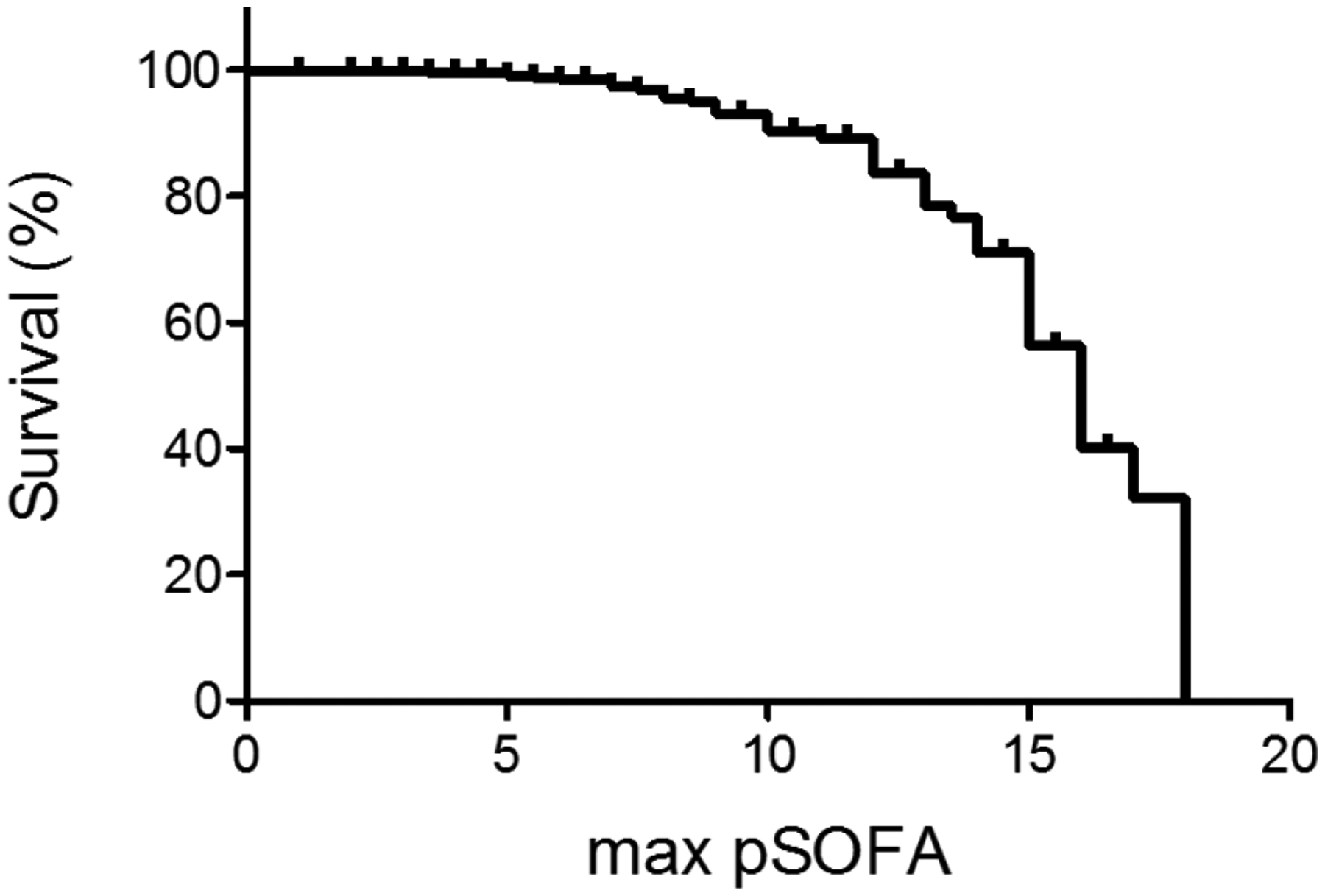
The relationship between sepsis survival (%) and maximum pSOFA score.

Figure 1 demonstrated the relationship between maximum pSOFA score and survival. Higher maximum pSOFA correlated with higher mortality (Table 4). The cutoff value to predict mortality was 11 (Area under the curve (AUC) of 0.720).

**Figure 2:**
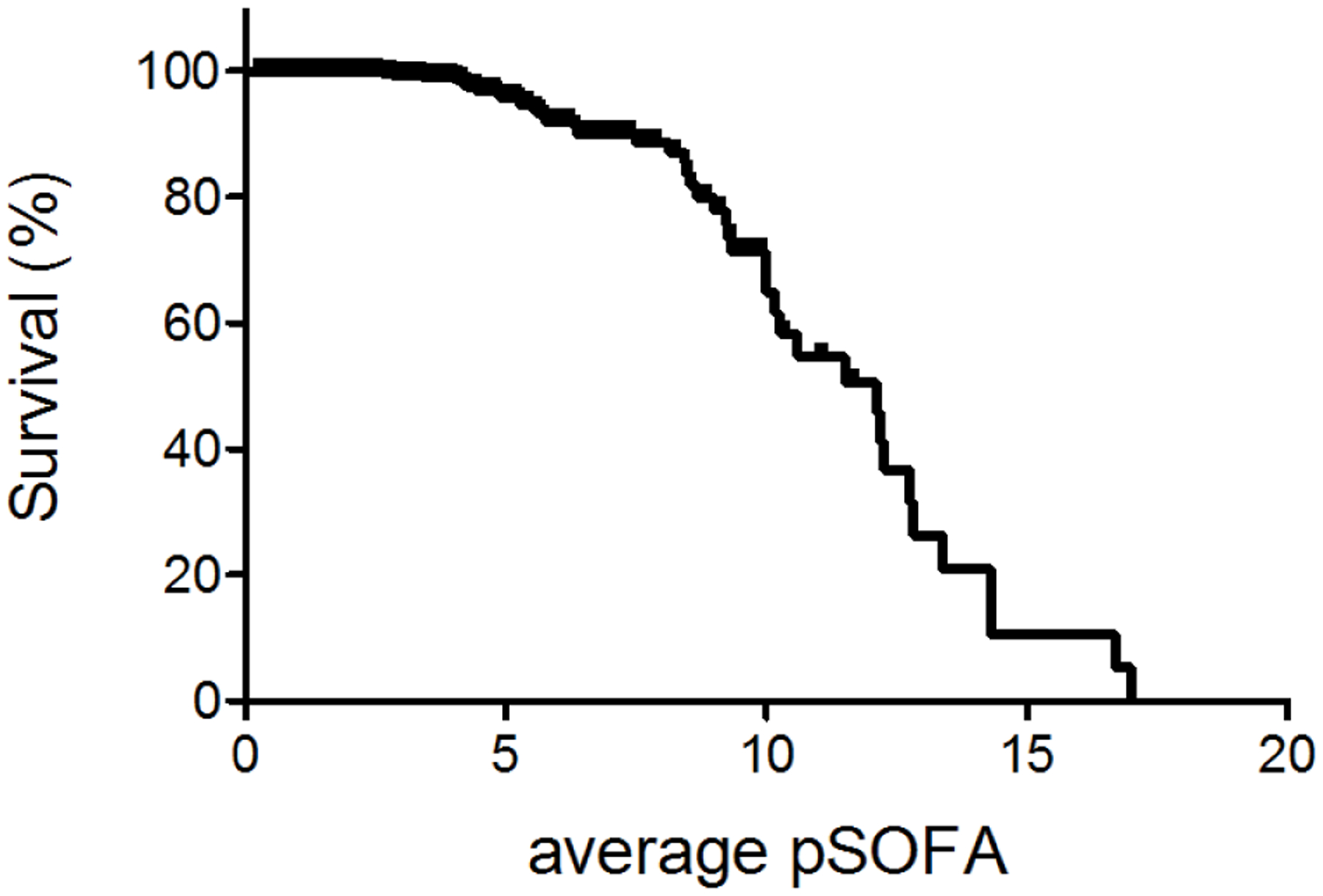
The relationship between sepsis survival (%) and average pSOFA score.

Figure 2 described the relationship between average pSOFA and survival. Higher average pSOFA correlated with higher mortality (Table 5). The cutoff value to predict was 5 (AUC of 0.750).

### The type of microbes

Fewer patients were associated with detectable microbes (Table 3). Identification of gram-positive and/or gram-negative bacteria was associated with an increase in mortality. Table 6 described the type of detected microbes. *Staphylococcus aureus* was the most common gram-positive bacteria and *Pseudomonas aeruginosa* was the most common gram-negative bacteria. In contrast, patients without any microbe identification were associated with better survival (Table 3).

## Discussion

Sepsis is a dysregulated host response to infection. The Sepsis, Prevalence, Outcomes, and Therapies (SPROUT) study was the first worldwide prospective study of pediatric sepsis and was reported in 2015. In this study, the hospital mortality of severe pediatric sepsis was examined. The mortality rate was 25%, similar to the rate reported in the septic adult population [6]. This study also demonstrated an important issue related to sepsis diagnosis. The study used severe sepsis defined in the Sepsis-2 conference for inclusion. However, there is no specific metric to determine organ injury such as SOFA. This study showed only a moderate level of agreement in the diagnosis of severe sepsis among physicians [7–9]. Thus, the use of a reliable organ dysfunction/injury assessment tool is enthusiastically sought for [10].

Three different pediatric versions of the SOFA score system have been proposed so far [5,11,12]. Some difference exists among them such as cardiovascular system criteria (for example, use mean arterial blood pressure vs. systolic blood pressure) [13]. The criteria proposed by Matics, et al. have been tested in their institutional data. In the cohort, patients who met the Sepsis-3 criteria had a mortality of 12.1%. The reason why the mortality in the SPROUT study was much higher than Matics’ is unclear, but it would be difficult to point out based on potential differences in the assessment method of organ injury as described above. So far, these pSOFA criteria were applied by several groups. Zhao, et al. examined the data of pediatric patients in three pediatric ICUs in China from 2011 to 2019 [14]. Their mortality rate was 23.4%. The pSOFA score at 24 hours after admission was compared with SIRS. The pSOFA score had a better prediction of mortality than SIRS. Lalitha, et al. examined a pediatric cohort in a pediatric ICU in India from 2017 to 2019 [15]. Their mortality rate was 17.5%. pSOFA scores on day 1 and day 3 presented significant predictions of mortality. A similar conclusion was reported by El-Mashad, et al., who studied pSOFA score on day 1 in Egypt in 2018 [16]. The SPROUT study showed that the mortality differed across geographic regions; the mortality rate was 21% in North America, 29% in Europe, 32% in Australia/New Zealand, 40% in Asia, 11% in South America, and 40% in Africa. This could be due to many factors such as underlying diseases and medical infrastructure etc. This also indicates that it is important to validate the pSOFA score system in a different North American cohort other than Matics’. Our motivation to test the pSOFA score system in our cohort is because our institution is one of the major pediatric tertiary institutions. In this study, we also found that the pSOFA value better correlated with mortality in our pediatric data, further supporting the utility of the pSOFA system-driven sepsis diagnosis. In-hospital mortality of our cohort was 8.0% (46/572) when we restricted to patients who met Sepsis-3 criteria. In our original cohort (all the patients who were diagnosed with sepsis from 2014 to 2019), the mortality was 7.2% (46/641).

As a higher (p)SOFA value was associated with higher mortality, it would be important to determine if there is any specific cutoff value to predict mortality. Matics’ cohort used the maximum pSOFA score to determine the cutoff for mortality as in the adult sepsis study by Singer, et al. [3]. In both Singer and Matics studies, the cutoff was 8 [3,5]. Our cohort’s maximum pSOFA cutoff value to predict mortality was 11 (AUC of 0.720). The difference can be attributed to many factors including potentially different medical management and patient characteristics between the two institutes. In addition, we examined the average pSOFA. The cutoff of average pSOFA to predict mortality was 5 with the AUC of 0.750. To understand the overall course, average pSOFA may also be valuable. The underlying causes of pediatric sepsis differ. In the SPROUT study, the most common primary sites of infection were respiratory (40.2%), primary bloodstream (9.1%), followed by abdominal (8.3%) [6]. It would be interesting to determine if there would be different cutoff values to predict mortality depending on underlying diseases.

As described above, Schlapbach, et al. reported the pSOFA score system differently from Matics, et al. They examined their score system in Australian and New Zealand cohorts [11]. Similar to Matics’ system, higher SOFA values highly predicted mortality. Thus far, there is no report comparing three different pSOFA score systems in the same cohort. To use the same criteria worldwide, it would be important to compare them to select a more specific and sensitive version in the future.

One of the interesting findings in our study is that patients who did not have identified microbes were associated with better survival. Phua, et al. also reported that the culture-negative group had fewer comorbidities, milder severity of illness, shorter hospitalizations, and lower mortality in the adult cohort [17]. Huang, et al. reported that a culture-positive pediatric cohort was associated with longer hospital stays [18]. Similarly, the pathogen type was associated with longer ventilator days and ICU days in the pediatric cohort reported by Salud, et al. [19]. This may throw an interesting question if there would be a difference in pathophysiology between patients with identified microbes and ones who did not.

Our study has limitations. In addition to being a retrospective study, we did not examine patients who did not receive a sepsis diagnosis during the same admission using the Sepsis-3 definition. Therefore, we could have missed patients who did not meet the Sepsis-2 definition but met the Sepsis-3 definition. An additional issue is on sepsis diagnosis. Sepsis diagnosis was discretionally made by the ICU physician who cared for the patient, and whether each physician used consistent diagnosis criteria is unclear. Those concerns will be addressed by performing this type of study prospectively.

In conclusion, we showed that our single institution data supported the idea that pSOFA value better correlated with mortality. In the future, we need to determine the relationship between mortality and pediatric sepsis cohort using the Sepsis-3-based definition worldwide.

## Figures and Tables

**Table 1:** Pediatric SOFA scoring system.

	Score				
**Variables**	0	1	2	3	4
**Respiratory**					
PaO_2_/FiO_2_	> 400	300–399	200–299	100–199	< 100
or					
SpO_2_/FiO_2_	> 292	264–291	221–264	148–220	< 148
**Coagulation**					
Platelet (x10^3^/μL)	> 150	100–149	50–99	20–49	< 20
**Hepatic**					
Bilirubin (mg/dL)	< 1.2	1.2–1.9	2.0–5.0	6.0–11.9	> 12.0
**Cardiovascular**					
MAP					
< 1 mo	> 46	< 46	DOA < 5	DOA > 5	DOA > 15
1–11 mo	> 55	< 55	DOB < 5	EPI < 0.1	EPI > 0.1
12–23 mo	> 60	< 60		NOR < 0.1	NOR > 0.1
24–59 mo	> 62	< 62			
60–143 mo	> 65	< 65			
144–216 mo	> 67	< 67			
> 216 mo	> 70	< 70			
**Neurologic**					
GCS	15	13–14	10–12	6–9	< 6
**Renal**					
Creatinine (mg/dL)					
< 1 mo	< 0.8	0.8–0.9	1.0–1.1	1.2–1.5	> 1.6
1–11 mo	< 0.3	0.3–0.4	0.5–0.7	0.8–1.1	> 1.2
12–23 mo	< 0.4	0.4–0.5	0.6–1.0	1.1–1.4	> 1.5
24–59 mo	< 0.6	0.6–0.8	0.9–1.5	1.6–2.2	> 2.3
60–143 mo	< 0.7	0.7–1.0	1.1–1.7	1.8–2.5	> 2.6
144–216 mo	< 1.0	1.0–1.6	1.7–2.8	2.9–4.1	> 4.2
> 216 mo	< 1.2	1.2–1.9	2.0–3.4	3.5–4.9	> 5

mo: month; MAP: Mean Arterial Pressure; GCS: Glascow Coma Score

DOA: Dopamine; DOB: Dobutamine; EPI: Epinephrine; NO: Norepinephrine

**Table 2:** The comparison between pediatric patients who met sepsis-3 criteria and who did not.

	Sepsis-3 (n = 572)	Non-sepsis-3 (n = 69)	p value	95%CI	OR
Age (year)	6.0 (1.0, 13.0)	5.0 (2.0, 11.5)	0.350		
Gender	Male 290 (50.7%) Female 282 (49.3%)	Male 39 (56.5%) Female 30 (43.5%)	0.360	0.47–1.31	0.791
Weight (kg)	22.15 (11.43, 47.00)	18.80 (11.75, 37.20)	0.288	0.99–1.02	1.006
Average cardiovascular score	1.0 (0.67, 1.50)	0.67 (0.33, 1.00)	< 0.001	n/a	
Average respiratory score	2.37 (1.15, 2.94)	0 (0, 0)	< 0.001	n/a	
Average coagulation score	0.32 (0, 1.33)	0 (0, 0)	< 0.001	n/a	
Average hepatic score	0 (0, 0.03)	0 (0, 0)	< 0.001	n/a	
Average renal score	0 (0, 0.09)	0 (0, 0)	< 0.001	n/a	
Average pSOFA score	4.00 (2.81, 5.41)	0.67 (0.42, 1.00)	< 0.001	n/a	
Max cardiovascular score	2 (1, 3)	1 (1, 1)	< 0.001	3.44–9.06	5.584
Max respiratory score	4 (2.5, 4)	0 (0, 0)	< 0.001	n/a	
Max coagulation score	1 (0, 3)	0 (0, 0)	< 0.001	3.29–138.74	21.373
Max hepatic score	0 (0, 1)	0 (0, 0)	< 0.001	n/a	
Max renal score	0 (0, 1)	0 (0, 0)	< 0.001	1.13–16.20	4.280
Max pSOFA score	6 (4.5, 9)	1 (1, 1)	< 0.001	n/a	
Length of ICU stay	4.25 (2.02, 11.61)	1.02 (0.70, 1.72)	< 0.001	2.16–4.38	3.078
Number of death	46 (8.0%)	0 (0%)	< 0.001	n/a	

OR: Odds Ratio; CI: Confidence Interval; Max: Maximum; ICU: Intensive Care Unit; n/a, not available

**Table 3:** The comparison between pediatric patients who survived and who did not.

	Survivors (n = 641)	Non-Survivors (n = 46)	p value	95%CI	OR
Age (year)	6.0 (2.0, 13.0)	6.0 (1.0, 13.25)	0.680	0.94–1.04	0.989
Gender	Male 22 (47.8%) Female 24 (52.2%)	Male 307 (47.9%) Female 334 (52.1%)	0.622	0.47–1.57	0.860
Weight (kg)	21.90 (11.60, 46.90)	19.85 (10.08, 43.65)	0.670	0.99–1.02	1.003
Average cardiovascular score	1.00 (0.63, 1.36)	1.38 (0.75, 2.38)	< 0.001	1.71–3.50	2.446
Average respiratory score	2.03 (0, 2.75)	2.18 (2.72, 3.68)	< 0.001	2.63–6.97	4.280
Average coagulation score	0 (0, 1.00)	1.77 (0.38, 3.00)	< 0.001	1.76–2.84	2.235
Average hepatic score	0 (0, 0)	0.55 (0, 1.74)	< 0.001	2.51–5.12	3.583
Average renal score	0 (0, 0)	0.16 (0, 1.47)	< 0.001	1.57–2.76	2.080
Average pSOFA score	3.63 (2.00, 4.86)	8.44 (2.38, 8.44)	< 0.001	1.46–1.85	1.646
Max cardiovascular score	1 (1, 3)	3 (2, 4)	< 0.001	1.64–2.96	2.204
Max respiratory score	2.5 (0, 4)	4 (4, 4)	< 0.001	1.61–3.92	2.508
Max coagulation score	0 (0, 2)	3 (2, 4)	< 0.001	1.50–2.32	1.869
Max hepatic score	0 (0, 0)	0 (0, 2)	< 0.001	1.68–2.62	2.098
Max renal score	0 (0, 0)	1.5 (0, 3)	< 0.001	1.50–2.22	1.827
Max pSOFA score	5.5 (3.5, 8.0)	12.0 (8.0, 15.0)	< 0.001	1.29–1.52	1.399
Gram positive bacteria	16 (34.8%)	130 (20.3%)	0.047	1.01–3.61	1.908
Gram negative bacteria	16 (34.8%)	126 (27.3%)	0.035	1.05–3.76	1.985
Yeast	6 (13.0%)	32 (6.9%)		n/a	
Suspected undefined	17 (37.0%)	370 (80.3%)	0.001	0.19–0.66	0.356
Length of ICU stay	3.23 (1.69, 9.72)	6.79 (3.03, 34.47)	0.010	1.003–1.02	1.010

OR: Odds Ratio; CI: Confidence Interval; Max: Maximum; ICU: Intensive Care Unit; n/a, not available

**Table 4:** The relationship between maximum pSOFA and mortality.

Max pSOFA score	Number of patients	Death	Mortality percentage
0–5	299	4	1%
5 < - 10	249	17	7%
10 < −15	78	17	22%
15 < −20	15	8	53%

**Table 5:** The relationship between average pSOFA and mortality.

Average pSOFA score	Number of patients	Death	Mortality percentage
0 – 5	472	12	3%
5 < - 10	149	20	13%
10 < −15	18	12	67%
15 < −20	2	2	100%

**Table 6:** Type of microbes detected in pediatricseptic patients

Type of Infection	Organism	Number of Patients Infected (%)
Gram-Positive	*Staphylococcus aureus*	67 (10.5%)
Gram-Positive	*Enterococcus faecalis*	22 (3.4%)
Gram-Positive	*Enterococcus cloacae*	13 (2.0%)
Gram-Negative	*Pseudomonas aeruginosa*	46 (7.2%)
Gram-Negative	*Klebsiella pneumoniae*	23 (3.6%)
Gram-Negative	*Escherichia coli*	16 (2.5%)
Yeast	*Candida Albicans*	19 (3.0%)
Yeast	*Candida parasilosis*	7 (1.2%)
Yeast	*Candida krusei*	3 (0.5%)

## References

[1] Bone RC, Definitions for sepsis and organ failure and guidelines for the use of innovative therapies in sepsis. The ACCP/SCCM consensus conference committee. american college of chest physicians/society of critical care medicine. Chest 101: 1644–1655, doi:10.1378/chest.101.6.1644 (1992). 1303622

[2] Levy MM, 2001 SCCM/ESICM/ACCP/ATS/SIS international sepsis definitions conference. Crit Care Med 31: 1250–1256, doi:10.1097/01.CCM.0000050454.01978.3B (2003). 12682500

[3] Singer M, The third international consensus definitions for sepsis and septic shock (Sepsis-3). JAMA 315: 801–810, doi:10.1001/jama.2016.0287 (2016). 26903338 PMC4968574

[4] Vincent JL, The SOFA (Sepsis-related Organ Failure Assessment) score to describe organ dysfunction/failure. On behalf of the working group on sepsis-related problems of the european society of intensive care medicine. Intensive Care Med 22: 707–710, doi:10.1007/BF01709751 (1996). 8844239

[5] Matics TJ, Sanchez-Pinto LN. Adaptation and validation of a pediatric sequential organ failure assessment score and evaluation of the Sepsis-3 definitions in critically ill children. JAMA Pediatr 171: e172352, doi:10.1001/jamapediatrics.2017.2352 (2017). 28783810 PMC6583375

[6] Weiss SL, Global epidemiology of pediatric severe sepsis: The sepsis prevalence, outcomes, and therapies study. Am J Respir Crit Care Med 191: 1147–1157, doi:10.1164/rccm.201412-2323OC (2015). 25734408 PMC4451622

[7] Piva JP, Garcia PC. Sepsis: From the stone age to nowadays without a precise definition. Pediatr Crit Care Med 17: 794–795, doi:10.1097/PCC.0000000000000885 (2016). 27500615

[8] Weiss SL, Discordant identification of pediatric severe sepsis by research and clinical definitions in the SPROUT international point prevalence study. Crit Care 19: 325, doi:10.1186/s13054-015-1055-x (2015). 26373923 PMC4572676

[9] Weiss SL, Defining pediatric sepsis by different criteria: Discrepancies in populations and implications for clinical practice. Pediatr Crit Care Med 13: e219–226, doi:10.1097/PCC.0b013e31823c98da (2012). 22460773

[10] Kawasaki T Update on pediatric sepsis: A review. J Intensive Care 5: 47, doi:10.1186/s40560-017-0240-1 (2017). 28729906 PMC5518149

[11] Schlapbach LJ, Straney L, Bellomo R, MacLaren G, Pilcher D. Prognostic accuracy of age-adapted SOFA, SIRS, PELOD-2, and qSOFA for in-hospital mortality among children with suspected infection admitted to the intensive care unit. Intensive Care Med 44: 179–188, doi:10.1007/s00134-017-5021-8 (2018). 29256116 PMC5816088

[12] Shime N, Kawasaki T, Nakagawa S. Proposal of a new pediatric sequential organ failure assessment score for possible validation. Pediatr Crit Care Med 18: 98–99, doi:10.1097/PCC.0000000000001009 (2017). 28060166

[13] Kawasaki T, Paediatric sequential organ failure assessment score (pSOFA): A plea for the world-wide collaboration for consensus. Intensive Care Med 44: 995–997, doi:10.1007/s00134-018-5188-7 (2018). 29704146

[14] Zhao C, Comparing the precision of the pSOFA and SIRS scores in predicting sepsis-related deaths among hospitalized children: a multi-center retrospective cohort study. World J Emerg Med 13: 259–265, doi:10.5847/wjem.j.1920-8642.2022.060 (2022). 35837567 PMC9233967

[15] Lalitha AV, Sequential organ failure assessment score as a predictor of outcome in sepsis in pediatric intensive care unit. J Pediatr Intensive Care 10: 110–117, doi:10.1055/s-0040-1714705 (2021). 33884211 PMC8052111

[16] El-Mashad GM, El-Mekkawy MD, Zayan MH. Paediatric sequential organ failiure assessment (pSOFA) score: A new mortality prediction score in the paediatric intensive care unit. An Pediatr (Engl Ed) 92: 277–285 (2020). 31784324 10.1016/j.anpedi.2019.05.018

[17] Phua J, Characteristics and outcomes of culture-negative versus culture-positive severe sepsis. Crit Care 17: R202, doi:10.1186/cc12896 (2013). 24028771 PMC4057416

[18] Huang H, Chen J, Dang H, Liu C, Fu YQ. Comparing outcomes between culture-positive and culture-negative septic shock in a PICU: A retrospective cohort study. Front Pediatr 10: 1001565, doi:10.3389/fped.2022.1001565 (2022). PMC9608626 36313890

[19] Salud D, Association of pathogen type with outcomes of children encountering community-acquired pediatric septic shock. Pediatr Crit Care Med 23: 635–645, doi:10.1097/PCC.0000000000003001 (2022). 35687094 PMC9529775

